# Ranking Biomedical Annotations with Annotator's Semantic Relevancy

**DOI:** 10.1155/2014/258929

**Published:** 2014-05-11

**Authors:** Aihua Wu

**Affiliations:** Department of Computer Science, Shanghai Maritime University, Shanghai 201306, China

## Abstract

Biomedical annotation is a common and affective artifact for researchers to discuss, show opinion, and share discoveries. It becomes increasing popular in many online research communities, and implies much useful information. Ranking biomedical annotations is a critical problem for data user to efficiently get information. As the annotator's knowledge about the annotated entity normally determines quality of the annotations, we evaluate the knowledge, that is, semantic relationship between them, in two ways. The first is extracting relational information from credible websites by mining association rules between an annotator and a biomedical entity. The second way is frequent pattern mining from historical annotations, which reveals common features of biomedical entities that an annotator can annotate with high quality. We propose a weighted and concept-extended RDF model to represent an annotator, a biomedical entity, and their background attributes and merge information from the two ways as the context of an annotator. Based on that, we present a method to rank the annotations by evaluating their correctness according to user's vote and the semantic relevancy between the annotator and the annotated entity. The experimental results show that the approach is applicable and efficient even when data set is large.

## 1. Introduction

Annotations are allowed in most online biomedical databases like NCBI (http://www.ncbi.nlm.nih.gov/), UCSC Gene Browser (http://genome.ucsc.edu/), GDB (http://www.gdb.org/), DDBJ (http://www.ddbj.nig.ac.jp/), and so forth. Shared annotations are becoming increasingly popular in online communities. It is a fundamental activity and plays an important role in the normal research community, with which researchers can explain and discuss the experimental data and share their discoveries [1–5]. As shared comments on documents, pictures, videos, and other annotations, it is also an important data source for biomedical researcher, because of its implying additional facts and annotator's opinions about the biomedical entity. As an example, researchers discovered information about a new protein family with annotations in Flybase [6] and UniProtKB/Swiss-Prot [7]. Now, more and more researchers recognize that it is important to attach and analyse annotations on biomedical entities.

As an open community, there may be many annotations attached with a single biomedical entity. Thus, a question of how to rank the annotations so that users can spend the least time to get the most useful information arises.

Ranking annotations is important and useful for an online biomedical community. As known, biomedical research is active and knowledge about the biomedical entity can be renewed every day. Many of the new discoveries appear in form of annotations. To follow the latest thinking and discovery, researchers will spend much time to view these annotations. A ranking module can help them to retrieve high quality annotations quickly and improve efficiency of the discussions. Rankings also encourage users to publish correct and validated opinion and materials about the biomedical data, so that the community will be more active and become a more important data center and discussion platform.

Ranking reviews, which can be viewed as a type of annotation, are a common problem in many e-commerce and news websites [8, 9]. Popular previous methods are mostly based on voting or scoring. Unfortunately, voting and scoring cannot avoid spreading wrong opinions, because users would like to agree with the most popular reviews, even if they do not know whether it is right or not. As a result, useless, even spiteful, reviews constantly appear in the top position in many websites.

As we know, quality of a scientific annotation depends in part on how much the annotator learns about the biomedical entity. The more knowledge the annotator has, the more correct his annotations can be, thus, the more useful to the data user. For example, as for the H1N9 virus, annotations from an astrophysicists are normally with lower correctness than those submitted by a biologist who concentrates on bird flu. User's knowledge is indicated by his semantic background such as working experience, study, and research. If given user is viewed as an object, the semantic background will be a composite of all attributes that describe the user or his related objects and so the biomedical entity can be described. We say that a biomedical entity and a user are semantic related if their semantic backgrounds are partly matched. Obviously, the more they matched, the more the user may learn about the entity.

In the scientific community, an obvious fact is that the annotator's knowledge can be reflected in papers he published and approaches he focused on, which can be obtained from the Internet or other public data source. With such background data, how the annotator may learn about the entity he annotated can be deduced. Besides, accepted historical annotations do also reflect the annotator's knowledge about the annotated entity. If an annotator always contributes high quality annotation to entities with the same attributes, we can say he is familiar with other such entities. In this paper, we propose a weighted and concept-extended resource description framework (RDF) [10] to represent an annotator and a biomedical entity. For any given pair of annotator and biomedical entity, a RDF graph will be created, where the annotator is the root node, attributes of the entity and its one-step extended concepts are the leaf nodes, and each edge is assigned a weight denoting how much the root node learns about the target node. The weight will be evaluated by their cooccurrence in credible web data. On the other hand, frequent patterns of the biomedical entities that was historically annotated by given annotator will be mined. Suppose there is no malicious user, people only annotate biomedical entity that they know. Both the weight and the matching degree of the annotated entity to the frequent patterns are explained as the semantic relevancy. Accordingly, we present a method to rank the annotations by evaluating their correctness with the semantic relevancy between the annotator and the biomedical entity.

*Organization*.  Section 2 is related works.  Section 3 introduces the weighted RDF graph model and related concepts.  Section 4 presents two main works of this paper. One is how to initialize RDF graph of an annotator and a biomedical entity by web information extraction, including details of computing weight for an annotator's RDF by association mining open credible web information. The other is the algorithm for mining frequent item of historical annotated biomedical entities.  Section 5 shows formulas evaluating correctness of a new annotation.  Section 6 states experimental results. And last section is the conclusion.

## 2. Related Work

Evaluating and ranking biomedical annotations are new problems. The most similar researches are ranking reviews, estimating quality of web content, and opinion strength analysis.

Ranking reviews or other web content has always been a complex problem and attracts renewed research interests in many fields, especially as web plays an increasing important role in delivering and achieving information for many people. Most previous methods are based on user's reputation, word-of-mouth, webpage links, and the other types of user's voting [8, 9, 11–14]. Ai and Meng proposed a method based on weighted fan-in page links and copies to recommend recruitment advertising [11]. It has a viewpoint that the more the users believe and the more dependable the websites are, the higher the quality of the advertisement will be. Largillier et al. present a voting system for news articles using statistical filter and a collusion detection mechanism in [8]. It is reasonable to rank web content according to author's reputation and user's voting in some applications. The former is unworkable when the user does not have enough historical annotations, while the latter cannot exclude propagation of rumors. In this paper, we try to evaluate annotation's quality from the new perspective of the semantic relation between annotators and the annotated biomedical entities, which is, to the best of our knowledge, scarcely considered by previous approaches. In biomedical domain, correctness of user's annotations largely depends on annotator's knowledge about the annotated entities. Semantic relevancy between them plays a critical role in the quality evaluation. Our method is more convincing.

Some prior works try also to discover inherent relationship between data and its users by data mining techniques [15–22]. They can be classified into three categories: statistical methods based on cooccurrence of terms [16], machine learning techniques [17], and hybrid approaches of them [18]. Staddon and Chow studied online book reviews of http://www.amazon.com/ and proposed a method of quality evaluation by mining the association rules between book authors and book reviewers [15]. In [22] the authors proposed three models to evaluate quality of Wikipedia articles by measuring the influence of author's authority, review behavior, and the edit history on quality of the article. These researches also try to discover semantic relationship between data and its users, but they did not consider textual content of reviews or other online opinions [18–20], and their criteria are simple; for example, association relationships are defined as the cooccurrence of the author's name and the annotator's name on web in [15]; as a result, they cannot reveal comprehensive semantic relevancy. We describe the entities by their entire semantic context with their attributes and related biomedical entities and based on that, we can analyze multidimensional semantic relationships between biomedical entities and their annotator. Still, we parse the textual content of the annotation and highlight attributes mentioned in it when matching patterns and evaluating its correctness.

Other related works are biomedical web information extraction, biomedical text mining, and biomedical entity recognition [23–28]. They are related but independent problems. We did not propose new algorithms for those problems and we did not develop a related tool, but we applied existing methods and applications. You can find some performance trials on the website of the Biocreative group (http://www.biocreative.org/) [23], ontology-driven term extraction service for biomedical text on the National Center for Biomedical Ontology (NCBO), and biomedical text mining applications developed by several academic groups and other organizations [24–29].

## 3. Weighted RDF Graph and Concepts

**Figure 1:**
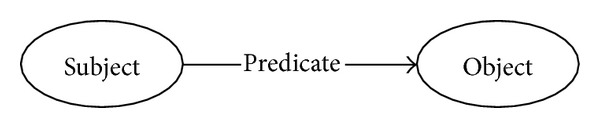
The atom triple of RDF.

RDF is a graph based framework for representing concepts on the web by linking its concrete syntax to its formal semantics. In RDF, any expression is a triple of a subject, a predicate, and an object, which can be illustrated by a node-arc-node linked graph as shown in Figure 1. Node represents a subject or an object, and directed arc with a predicate represents relationship between them.

**Figure 2:**
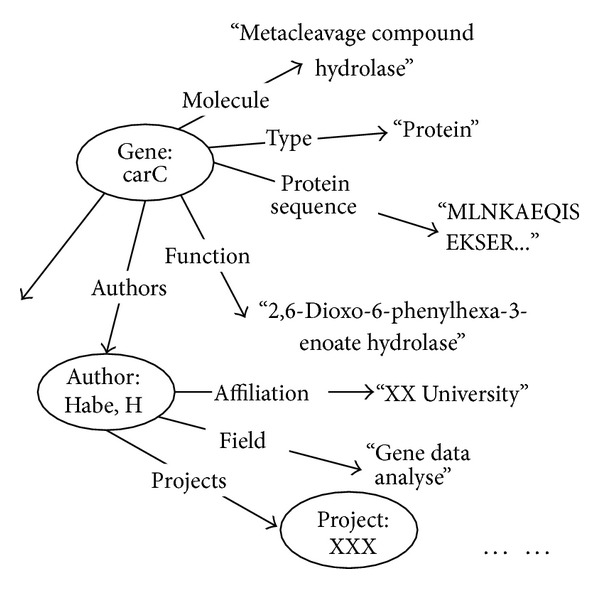
RDF graph of a biomedical entity.

A biomedical entity can be viewed as a RDF subject; its attributes and concept field can be looked at as its objects.  Figure 2 shows the RDF graph of protein structure 1J1I in RCSB, whose main features include molecule, protein sequence, function, and authors. Attribute nodes can be extracted from the online biomedical databases and their linked credible web sites. Here, we say that a node is an attribute node if its outdegree is 0, and the others are* entity nodes*. Tag of an* entity node* is composed of type name and ID of the entity in form of* typeName*:*entityID*. Attributes nodes will be extracted as more as we can so that an entity can be specified more exactly.

An annotator can also be viewed as a RDF subject, and biomedical entities he/she annotated can be its objects. Annotators may have many attributes, but we only consider those locally described and those related to the biomedical entity. We use two types of RDF graph to specify an annotator. One is named annotator's RDF graph whose composing details are present in Section 4.1, and the other is a set of frequent patterns of his/her historical annotated entities. In the RDF graph of annotator *P*, the annotator is the root node, the biomedical entity and its related concepts are the annotator's objects node, and weight on edge pointing to node *A*, which is marked as *ω*_*P*_^*A*^, is initialized as the correlation degree of *P* and *A*. Instead of weight, frequency and correctness are attached to each pattern, indicating their semantic relevancy.

Different from others, scientific data has complicated concept background. It can be a node in a complex relation network. There is a high possibility that people learning *A* will also learn about *A*'s subconcepts, *A*'s father concept, or *A*'s related concepts. For example, an annotator who knows many of* Trichophyton tonsurans* and* Trichophyton schoenleini* may also know about* Trichophyton rubrum*, because they all a type of mycosis causing similar tinea. Intern weight will be calculated for such possibility.

**Definition 1 (intent weight)**

Suppose annotator *u* learns about concept *A*_1_ with weight of *N*, *B* is a father concept or a related concept of *A*, and there are *M* − 1 other concepts *A*_2_, *A*_3_,…, *A*_*m*_ who are also *B*'s subconcept or related concept, but *u* does not indirectly know about them; then weight on edge pointing to *A*_*i*_(2 ≤ *i* ≤ *m*) in annotator graph of *u* is *N*/*M*. Such weight is called intent weight of *A*_*i*_ against *A*_1_, marked as *ϖ*_*P*_^*A*_1_…*A*_*i*_^.

Total intent weight *ϖ*_*u*_^*A*^ of a concept *A* in u's RDF graph is defined as follows:

(1) $$\begin{array}{c} \varpi_{u}^{A}=\overset{i<=N}{\underset{i=1}{\sum }}\left(\frac{\omega_{u}^{A_{i}}}{M_{i}}\right). \end{array}$$

Here, *A*_*i*_ is father or related concept of *A*, *M*_*i*_ is number of concepts whose relationship with *A*_*i*_ is identical to that of *A* with *A*_*i*_, and the relationships are defined in open biomedical databases such as FACTA+ and Go Terms.

**Definition 2 (RDF path)**

(1) If there is an edge *e* between an entity node *E* and an attribute node *A*, we say that *E*/*e*/*A* is a RDF path between *E* and *A*. (2) If there is a RDF path*p* between entity node *E*′ and *A* and an edge between entity node *E* and *E*′, we say that*E*/*p* is a RDF path between *E* and *A*. The first node is* root node* of a RDF path. And* pattern path* is a* RDF path* without entity node value.

In Figure 2, “Gene:carC*∖*type*∖*‘protein'” is a RDF path, and “Gene*∖*type*∖*‘protein'” is a pattern path.

**Definition 3 (prefix path)**

Given a RDF path or a pattern path *p*, the subsequence from the root node to edge pointing to a nonroot node *E* is a prefix path of *E* in *p*.

Two RDF paths with identical prefix path are* conjugate*. Conjugate RDF paths can be merged into a sub-RDF graph and conjugate sub-RDF graphs can be merged into a bigger sub-RDF graph when merging the identical ancestor nodes.

Given two RDF paths *p* and *g*, if there is a RDF path *p*′ in *g*, where *p*′ = *p*, we say that *p* ⊂ *g*. Similarly, Given two sub-RDF graphs *g*1 and *g*2, if, for all *p* ⊂ *g*1 (*p* is a RDF path), *p* ⊂ *g*2, we say that *g*1 ⊂ *g*2, and if *g*1 ⊂ *g*2 and *g*2 ⊂ *g*1, we say that *g*1 = *g*2.

Likewise, two pattern paths with same prefix path are* conjugate*. Two conjugate pattern paths can be merged into a subpattern RDF graph. And a pattern path can belong to a pattern RDF graph *g*, if it is equal to a path in the graph. And for any two pattern RDF graphs *g*1 and *g*2, if, for all *p* ⊂ *g*1 (*p* is a pattern path), *p* ⊂ *g*2, we say that *g*1 ⊂ *g*2, and if *g*1 ⊂ *g*2 and *g*2 ⊂ *g*1, we say that *g*1 = *g*2.

Additionally, let us define some symbols used as follows.

- *pp*_*u*_^cr,*f*^|^*N*=*n*^ is a frequent pattern path of user *u* from biomedical entity *O* to *N* with correctness cr and frequency *f* and*n* is value of attribute *N*. Similarly, *pp*_*u*_^*ϖ*^|^*N*=*n*^ is a path of user *u* pointing to *N* with weight *ϖ* and *n* is value of attribute *N*.
- *P*_*u*_^cr^ is a frequent pattern of user *u* on attribute *B* with correctness of cr, which is composed of frequent pattern paths.

## 4. Building Annotator's RDF Graph

In the following, Section 4.1 states details of composing annotator's RDF graph and computing weights by association mining open credible web information. And Section 4.2 presents frequent mining algorithm.

### 4.1. Initializing Annotator's RDF Graph with Web Information

Too much information can be extracted from the huge Internet, but only those of the biomedical entity and the annotation are useful in this application.

Given an annotation 〈*u*, *o*, *r*〉 where *u* is the annotator in form of a RDF node or a RDF graph, *o* is RDF graph of the biomedical entity, and *r* is the annotation, complete RDF graph of *u* is comprised of the following:

- *u*,
- *o*,
- an edge from the root node of *u* pointing to *o*.

Here (1)  *u* is initialized as an entity node when no local information can be used or a RDF graph generated according to the annotator's background data from the online database itself; (2)  *o* is initialized as stated in the following.

*Generating RDF Graph for a Biomedical Entity*. RDF graph of a biomedical entity *o* is initialized according to what is described in the online database. In our experiments, we created *o* by the following steps.

1. Recognize id (e.g., DOI) and type (protein, virus, etc.) of the biomedical entity with predefined keyword or normal structure and compose its entity node with tag of “Type:id.”
2. Extract each head item as an edge from predefined module such as “molecular description” and “experimental detail” and extract the value of the item as its attribute node or compose another level of entity nodes if the module contains several items and draw edges from the entity node to the attribute node.
3. Extract family classification according to the linked database on the page like Go Terms, look one step more into the detail of the linked database, recognize relationships between entities (e.g., mapping a protein to an organism or finding protein of the same family), draw RDF graph for them, merge the RDF graphs of different linked databases, and eliminate duplicate RDF paths.

**Figure 3:**
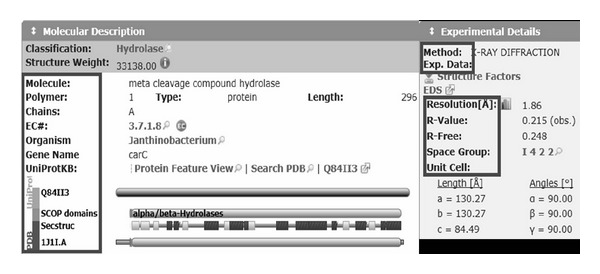
Examples of information extraction for annotated object.

**Figure 4:**
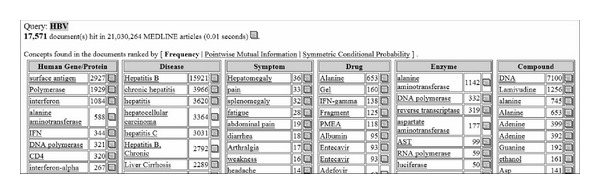
An example of one-step extension of the biomedical entity.

Figure 3 shows a segment of the information we will extract from the online database, and the circled items will be extracted as edge and their value will be extracted as attribute nodes.  Figure 4 shows an example of one-step extension of the biomedical entity's related concept to FACTA+.

*Annotation Analysis*. Bioconcepts in the annotations can be extracted by biomedical text analysis tools like GENIA [29] and the others. These concepts are normally the annotation's topic. We extract bioconcepts and their attribute names in an annotation; here the attributes names can be recognized by patterns “XX of bioconcept” or “bioconcept's XX.” For each concept, we draw an entity node and an edge for each of its attribute names even without attribute value. Merge and marked out the RDF graphs of the annotation into that of the biomedical entity *o*. If they cannot be merged, draw an edge from the annotator to its root nodes without weight.

*Weight Calculating*. We assign the weight on an edge will be assigned as the co-occurance of the annotator and the edge's target node in credible open data sources, such as news/talks/papers/personal pages published by predefined credible organizations, known proceedings, and websites. In the experiment, we use Google to search the news, talks, and personal pages, while Anne OTate [30] and PIE [31] to search papers on PubMED and MEDLine. At present, we did not consider the situation of different concepts inferring with the same biomedical entity, which is another scientific problem known as the biomedical text mining and clustering.

Suppose term of the annotator *u* is *t*1, term of the node *A* is *t*3, and term of the edge pointing to *A* is *t*2; then weight on the edge from web is defined as follows:

(2) $$\begin{array}{c} \omega 1_{u}^{A}=\{\begin{array}{cc} \frac{\left(c\left(t1\wedge t2\right)+c\left(t1\wedge t3\right)-c\left(t1\wedge t2\wedge t3\right)\right)}{c\left(t1\right)} & \\ \;\;A\;\;\text{is}\;\;\text{an}\;\;\text{attribute}\;\;\text{node} & \\ \sum \omega 1_{u}^{Bi} & \\ \;\;A\;\;\text{is}\;\text{not}\;\;\text{an}\;\;\text{attribute}\;\;\text{node}. & \end{array} \end{array}$$

Here *c*(*t*1∧*t*2) is the count of web pages that include *t*1 and *t*2, and *Bi* is an object node that *A* points to.

Considering the fact indicated by intent weight *ϖ*1_*u*_^*A*^, weight on the edge from web is finally defined as follows:

(3) $$\begin{array}{c} \omega 1_{u}^{A}=\omega 1_{u}^{A}+\varpi 1_{u}^{A}. \end{array}$$

### 4.2. Mining the Frequent Entity Patterns

Annotator's knowledge about a biomedical entity can also be inferred by his historical annotations. In this section, we will present an algorithm to discover frequent features of the historical annotated entities with correctness larger than 0.6. The algorithm will consider not only direct attributes of the entity, but also that of its one-step extended related concepts.

**Figure 5:**
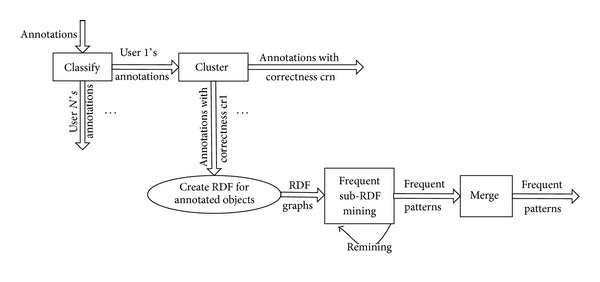
Illustration of the series of algorithms to mining frequent entity patterns.

As illustrated in Figure 5, firstly, the algorithms classify all annotations according to their annotator and then cluster each subset of annotations against their correctness with *K*-means. And correctness of each annotation in the cluster will be viewed as that of the cluster center. Lastly, frequent patterns are mined over biomedical entities in each cluster. Several questions arise here. First, because of the classification and cluster, the input data set can be too small to produce any patterns. The algorithms use* Laplacian* smoothing to solve it. Second, the algorithms can bring too much frequent patterns, while some of them can be included in or similar to another one. The algorithm uses Rule 1 to merge those that describe the same owner and the same attribute but with different attribute values and Rule 2 to merge the same patterns but with different correctness. Third, the data sets can be improperly clustered so that frequent pattern cannot be found. The algorithms use a new round of cluster and frequent pattern mining until mining results do not change.

**Figure 6:**
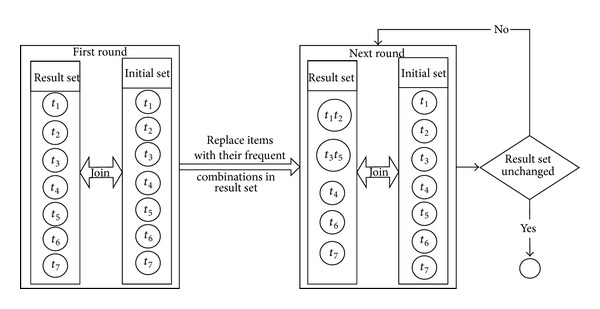
Illustration of frequent sub-RDF graphs mining.

Frequent sub-RDF graphs mining is the key step in the whole algorithm (step 2.3 of Algorithm 1). It takes the pattern paths of the entities as the items. Both the initial and final results are initialized as set of the frequent items obtained by the first round scan, and the result set is repeatedly refreshed by replacing each element with its one-item extension if the extension is also frequent. As shown in Figure 6, in the first round extension, each element in result set will conjunct with each element in initial set; for example, conjunctive of *t*_1_ and *t*_2_ is also frequent, so *t*_1_ and *t*_2_ will be replaced by *t*_1_ *t*_2_ in the result set.

**Rule 1**

Suppose that *p*1_*u*_^cr^, *p*2_*u*_^cr^,…, *pn*_*u*_^cr^ are a set of frequent patterns of user *u* with the same correctness rate cr and paths *pp*1^*N*1,*f*1^ ∈ *p*1_*u*_^cr^, *pp*2^*N*2,*f*2^ ∈ *p*2_*u*_^cr^,…, *pp* *n* ^*Nn*,*fn*^ ∈ *pn*_*u*_^cr^ with the same or different frequency; if *N*1, *N*2,…, *Nn* are different attribute values of the same attribute node *N*, then *pp* *i* ^*Ni*,*fi*^(1 ≤ *i* ≤ *n*) can be replaced by *pp* ^{*N*1,*N*2,…*Nn*},*f*^. Specially, if *p*1_*u*_^cr^, *p*2_*u*_^cr^,…, *pn*_*u*_^cr^ are only different with each other on *pp* *i* ^*Ni*,*fi*^(1 ≤ *i* ≤ *n*), then they can be merged into *p*_*u*_^cr^|_*N*  in{*N*1,*N*2,…*Nn*}_ and can replace *pp* *i* ^*Ni*^(1 ≤ *i* ≤ *n*) with *pp* ^{*N*1,*N*2,…*Nn*},*f*^; furthermore, if domain *N* = {*N*1, *N*2,…, *Nn*}, then they can be merged into *p*_*u*_^cr^|_*N*=any_ by replacing *pp* *i* ^*Ni*,*fi*^(1 ≤ *i* ≤ *n*) with *pp* ^any,*f*^. In each target path, frequency *f* = ∑_*i*=1_^*i*=*n*^ *fi*/*n*.

**Rule 2**

Suppose that *p*1_*u*_^cr1,*f*1^, *p*2_*u*_^cr2,*f*2^,…, *pn*_*u*_^cr*n*,*fn*^ are a series of frequent patterns of user *u* but with different correctness and the same or different frequency; if *p*1 = *p*2 = ⋯ = *pn*, then *p*1_*u*_^cr1,*f*1^, *p*2_*u*_^cr2,*f*2^,…, *pn*_*u*_^cr*n*,*fn*^ can be merged into *p*_*u*_^cr,*f*^, where cr = ∑_*i*=1_^*i*=*n*^(cr*i*∗*fi*)/∑_*i*=1_^*i*=*n*^ *fi* and *f* = ∑_*i*=1_^*i*=*n*^ *fi*/*n*.

## 5. Ranking Annotation

In this section, we propose an algorithm to evaluate correctness (quality) for an annotation *r*(*u*, *o*) of biomedical entity *o* from user *u* under different situations: (1)  *u* isdirect semantically related to *o*; (2)  *o* is an entity node in RDF graph of *u* or *o* matches at least one frequent entity pattern of *u* on *o*; (3)  *u* has annotated another biomedical entity which is similar to *o*; (4)  *o* has been annotated by other users who are similar to *u*; (5)  *u* has never annotated any entity and *o* has never been annotated. Obviously, annotator is semantic related to the annotated biomedical entity in the first two situations, especially 100% semantic relevant in the first one. We will give formulas to evaluate correctness of annotations for the two situations in Section 5.1., while problem of computing correctness in the last three situations is called a “new user” problem, which will be solved by borrowing the credibility of its nearest neighbor. And details will be stated in Section 5.2. Totally, annotations will be ranked decreasing according to evaluating results of all annotations on the biomedical entity.

Besides the semantic relationship, we also consider user's voting and historical annotations on similar annotated biomedical entities from similar annotators when computing credibility of annotations. User's voting is a direct parameter for the agreement degree. And for new user problem where no semantic relationship exists, similar historical annotations can be borrowed to estimate the annotation's correctness.

### 5.1. Evaluating When Semantic Related

When annotator *u* is an attribute node in the RDF graph of the biomedical entity *o* or *o* is an attribute node of *u*, we say that they are semantic related to each other. More strictly, for an annotation *r*(*u*, *o*), suppose *G*1 is RDF graph of annotation *r*, *G*2 is RDF graph of annotator *u*, *G*3 is RDF graph of biomedical entity *o*, and *Ω* is a set of frequent patterns of *u*, whose forming methods are all stated in Section 4; if ∃ a prefix path pr1 ∈ *G*3 and a prefix path pr ∈ *G*1 that pr1 = pr and one of *G*3'sentity node is *u*, we say that *u* is direct semantically related to *o*. Normally, if (1) there is a prefix path pr ∈ *G*1, where pr ∈ *G*2, or (2) there is at least a path in *G*3 matching a frequent pattern in *Ω*, we say that *u* is semantically related to *o*.

Given an annotation *r*(*u*, *o*), if user *u* is direct semantically related to biomedical entity *o* and supposing that *V* is a set of voting score on *r*, where only the max one of each user's voting will be kept, then correctness acr of *r* is

(4) $$\begin{array}{c} \text{acr}=1+\left(\frac{\sum v\left(v\in V\right)}{\left|V\right|}\right). \end{array}$$

Here, |*V*| is the number of the element in set *V*. Furthermore, suppose *G*1, *G*2, *G*3 is RDF graph of *r*, *u*, and *o* corresponding, and *Ω* is a set of frequent patterns of *u*, if *u* is non-directly but semantically related to *o*, correctness of *r* is decided by the weight of *G*1 in *G*3 and the max matching degree of *o* to a frequent pattern in *Ω*. Supposing that *P*_*u*_^cr^ isa frequent pattern of *u* with correctness cr andsupposing that *P*_*u*_^cr^ has *N* RDF pattern paths, among which *K* pattern paths (suppose *pp*1_*u*_^cr1,*f*1^,…, *pp* *K*_*u*_^cr*K*,*fK*^) match both a RDF path of *o* and a prefix path of *G*1, then the feature matching degree *d*_*o*_^*P*^ of *u* and *P*_*u*_^cr^ is defined as follows:

(5) $$\begin{array}{c} d_{o}^{P}=\overset{K}{\underset{i=1}{\sum }}\left(\text{c}{\text{r}}_{i}*f_{i}\right),\\ \text{c}{\text{r}}_{i},f_{i}\;\text{is}\;\;\text{correctness}\;\;\text{of}\;\;\text{pattern}\;\;\text{path}\;\;\text{p}{\text{p}}_{i}. \end{array}$$

And supposing that there are *M* paths of *G*1 belonging to *G*2 with weight *ϖ*1,…, *ϖM* on each edge pointing to the attribute nodes, then correctness acr of *r* is defined as follows:

(6) acr=max⁡(doP)+∑i=1Mϖi+(∑v(v∈V)|V|)(p∈Ω  and  p  match  a  prefix  path  of  G1).

### 5.2. Evaluating for “New User”

When there is neither annotator's RDF graph nor frequent patterns indicating that the annotator *u* and the entity *o* are semantically related, but *u* has annotated other biomedical entities or *o* has been annotated by other user, we can use the nearest neighbor to evaluate correctness of annotation *r*(*u*, *o*).

For a given biomedical entity *o*, its nearest neighbor is a set of biomedical entity in which each element *o*′ satisfies the next condition:

(7) $$\begin{array}{c} \frac{\left|pp_{2}\right|}{\left|pp_{o}\right|}>\varepsilon ,\;\;\frac{\left|pp_{2}\right|}{\left|ppo^{\prime }\right|}>\varepsilon . \end{array}$$

Here, |*pp*_2_| is number of RDF paths that belong to both o and *o*′, |*pp*_*o*_| is number of paths that belong to *o*, |*pp* *o*′| is number of paths that belong to *o*′, and *ε* is threshold defined by user.

Similarly, nearest neighbor of a given user *u* is also a set of users among which each user *u*′ satisfies the following conditions:

(8) $$\begin{array}{c} \frac{\left|\text{appear}\left(u,u^{\prime }\right)\right|}{\left|\text{appear}\left(u^{\prime }\right)\right|}>\varepsilon \;\text{or}\;\;\frac{\left|o^{\text{cr}>\theta }\right|}{\left|o^{\prime \text{cr}>\theta }\right|}>\varepsilon ,\;\;\frac{\left|o^{\text{cr}>\theta }\right|}{\left|o^{\prime \prime \text{cr}>\theta }\right|}>\varepsilon . \end{array}$$

Here, |appear(*u*′)| is number of unique appearance of *u*′ in papers, public talks, news, and so forth, especially papers in PubMED and MEDLine, while |appear(*u*, *u*′)| is the coappearance of *u* and *u*′ in the above data sources. |*o* ^cr>*θ*^| is number of biomedical entities that was annotated by both *u* and *u*′with correctness larger than user defined threshold *θ*, |*o* ^′cr>*θ*^| is number of biomedical entities that was annotated by *u* with correctness larger than user defined threshold *θ*, |*o* ^′′cr>*θ*^| is number of biomedical entities that was annotated by *u*′ with correctness larger than user defined threshold *θ*, and *ε* is threshold defined by user.

Now, given an annotation *r*(*u*, *o*), if user *u* is not semantically related to biomedical entity *o*, supposing that *V* is a set of unique user's voting score on *r*, supposing that *U* is a set of users who are the nearest neighbor of *u*, and *O* is a set of biomedical entity who are the nearest neighbor of *o*, then correctness acr of *r* is

(9) $$\begin{array}{c} \text{acr}=\{\begin{array}{cc} \left(\frac{\sum \text{ac}{\text{r}}_{u}^{oj}\left(oj\in O\right)}{\left|O\right|}\right)+\frac{\sum v\left(v\in V\right)}{\left|V\right|} & \\ \;\;O\;\;\text{is}\;\;\text{not}\;\;\text{empty} & \\ \left(\frac{\sum \text{ac}{\text{r}}_{ui}^{o}\left(ui\in U\right)}{\left|U\right|}+\frac{\sum v\left(v\in V\right)}{\left|V\right|}\right) & \\ \;\;O\;\;\text{is}\;\;\text{empty}\;\;\text{and}\;\;U\;\;\text{is}\;\;\text{not}\;\;\text{empty}. & \end{array} \end{array}$$

Here, |*V*| is also the number of the elements in set *V*. acr_*u*_^*oj*^ is correctness of annotation submitted by user *u* on biomedical entity *o*.

Lastly, given an annotation *r*(*u*, *o*), if user *u* never submits any annotation and biomedical entity *o* has never been annotated and supposing that *V* is a set of voting score on *r*, where only the max one of each user's voting will be kept, then its correctness acr is defined as

(10) $$\begin{array}{c} \text{acr}=\left(\frac{\sum v\left(v\in V\right)}{\left|V\right|}\right). \end{array}$$

## 6. Experimental Evaluation

There are three works in this paper: (1) extracting web information to compute relevancy of an annotator and a biomedical entity, (2) frequent pattern mining of the historical annotations, and (3) evaluating correctness of the annotations. We will state in this section how we use the existing tools to extract web information and get our experimental data and show performance of the frequent pattern mining and ranking evaluations.

### 6.1. Experimental Environment

Settings of the experiment are Intel Celeron 420 2.0 GHZ CPU, 1 GB memory, and windows XP+SP2. The local database is SQL Server 2000.

### 6.2. Data Preparation

As an example, we only use protein data in the experiments. But our approach can also be applied to other biomedical entities. We firstly get manually 500 protein structures and their scientific names from http://www.rcsb.org, download their files like FASTA sequence and PDB, crawl their web page, extract basic attributes from the files and webpage, and import them into SQL server. Then we search the Anne OTate [30] with scientific names of those protein structures and randomly get 1000 unique authors as our initial annotators. Although there are some annotations and ontology of biomedical entity in the online database, few of them are proper for the frequent pattern mining. Thus, we automatically generate 20000 historical annotations, of which 60 percent are designed as shown in Table 1 and the others are randomly generated: random annotator, random biomedical entity, and random annotation with random correctness.

As shown in Table 1 1000 of the annotators are classified as 9 types. Each type is designed to contribute certain number of annotations with correctness in certain range. To test the* cold-start* problem, several users are designed to contribute 5 or below annotations. On the other hand, to ensure the patterns can be found, at least five of each type of users will give annotations on 5 to 15 biomedical entities with common features.

As for the web information, we presearched and stored their weights in database for the 20000 pairs of users and biomedical entities. First, each biomedical entity will be one-step extended in FACTA+ to get its related concepts. Then, to evaluate the weight, we get information by two ways: searching Google for news, talks, and homepages and searching PIE the search [31] for papers and other documents. To search Google, we write a C# program which autosearches the predefined credible websites with Google service using keywords including name/affiliation of the annotator, scientific name of the biomedical entity, extended concept, or attribute name of the biomedical entity as a plus. On the other hand, we apply and evaluate PIE the search to count the documents that indicate their semantic relationship. The resulting corpus contains a set of medical articles in XML format. From each article we construct a text file by extracting relevant fields such as the title, the summary, and the body (if they are available).

### 6.3. Frequent Pattern Mining

**Figure 7:**
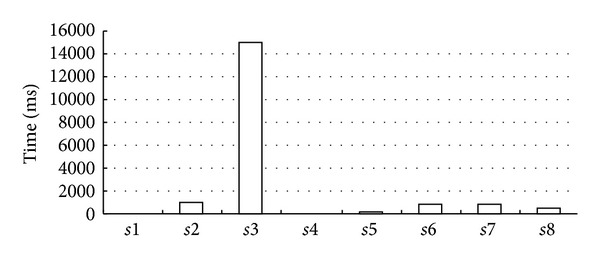
Time performance of frequent pattern mining.

We test 8 groups of data ($\begin{array}{c} s1\sim s8 \end{array}$ in Table 2), each of which only including annotations published by one annotator and belonging to one correctness group. The max group (*s*7) has 700 annotations and about 36 biomedical entities but on different attribute sets, while the min group (*s*3) has 100 annotations and about 10 biomedical entities. Biomedical entities in each group have some common attributes, which can be recognized as frequent pattern paths (fre. Attr. column in the table) after the first round of computing in the algorithm. Some of the frequent pattern paths appear in every biomedical entity, we say that they are 100% fre. Attr. Association of such items is certainly frequent; thus, we put their association directly into the finial mining result set but ignore another round of computing. The experimental results (Figure 7) show that the main time consumer is recursively computing the associate frequent pattern paths. *s*3 takes the highest time, because the 18 frequent (frequency below 100%) items need 15 rounds of computing to judge whether any level of their associations is also frequent. *s*4 is carried out at minimal cost, because no frequent pattern path can be found and only the first round of computing will happen.

### 6.4. Ranking

**Figure 8:**
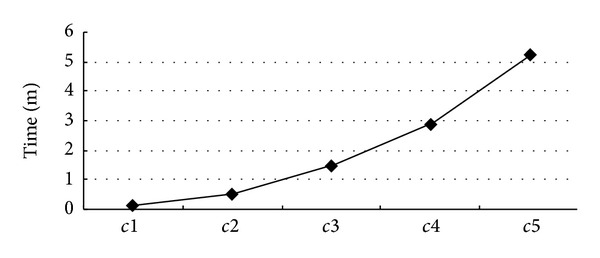
Time performance of correctness evaluation and ranking.

The experiments are executed over 5 sets of data. Different data sets contain different scales of annotations and frequent patterns. As shown in Table 3, *c*1 is the minimal data set, where 5000 annotations submitted by 100 annotators on 50 biomedical entities will be evaluated and ranked with 49 frequent patterns, while *c*5 is the maximal one including 40,000 annotations from 200 annotators on 200 biomedical entities, where it will be evaluated and ranked with 400 frequent patterns. For that weight on edge between each user and biomedical entity are precomputed and stored in database, the most time-consuming is the pattern matching. As shown in Figure 8, time goes up as number of patterns or annotations goes up. But even for *c*5, 5 minutes is enough to rank 40,000 annotations, which show the efficiency and applicability of the algorithm.

## 7. Conclusion

In this paper, we propose an approach for ranking biomedical annotations according to user's voting and semantic relevancy between an annotator and the biomedical entity he annotated. Our idea is inspired by the fact that in a credible online scientific community, quality of web content is determined to some extent by the contributor's knowledge about the entity. People's knowledge can be discovered from his profile and his related historical behaviors, especially for the researchers who are deeply specialized in one scientific domain. Thus, our major work in this paper is to find out how much a given annotator may learn about a biomedical entity from his profile on the web and frequent patterns of entities that he annotated in history.

An entity can be semantically defined by its attributes and its related entities' attributes. And people's knowledge about an entity can be reflected by the annotator's knowledge about those attributes. To express such relation, we extend the RDF model by assigning weight on each edge, which denotes the degree of how the root node (the annotator) knows about the target node (an entity or one of its attributes). The weight can be evaluated with the cooccurrence of the annotator and the target node in credible web information. Besides, an intent weight can indicate that people who know concept *A* may also know *A*'s related concept.

The second way to discover how the annotator semantically relates to the biomedical entity is frequent pattern mining over historical annotations, which revealed the common features of biomedical entities that an annotator may know. The pattern mining algorithm proposed in this paper can deal with problems caused by small example space, cold-start, and improper data source dividing.

In the future, we will go further on how to link record of a user and extract his profile information from the Internet when duplicate and uncertain data happen.

## Figures and Tables

**Algorithm 1:**
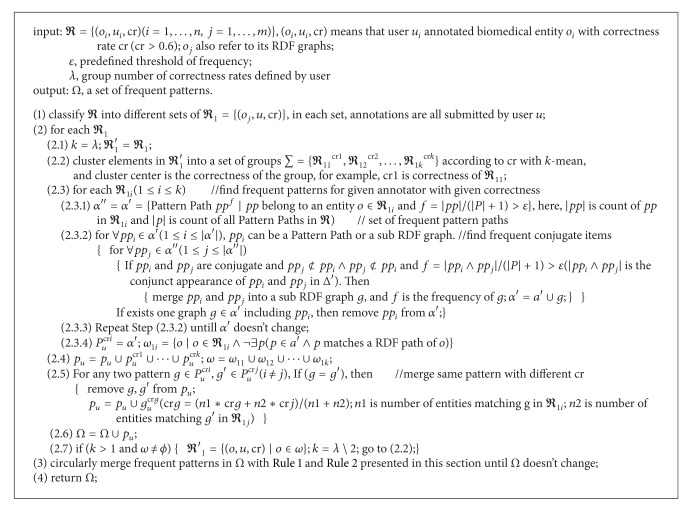
Frequent pattern.

**Table 1:** Annotator and annotation predefined in the experiments.

UserType/Num	Details of the designed annotations	Annotation ratio
U1/200	All annotation are 100% correct, and 5 of them only contribute 5 or below annotations	15%
U2/300	40% annotation with correctness 0.95~1; 50% annotations with correctness 0.9~0.95; 10% annotations with correctness 0.85~0.9; 22 of them only contribute 5 or below annotations	30%
U3/200	15% annotation with correctness 0.95~1; 55% annotations with correctness 0.9~0.95; 20% annotations with correctness 0.85~0.9; 10% annotations with correctness 0.8~0.85; 30 of them only contribute 5 or below annotations	15%
U4/80	10% annotations with correctness 0.9~0.95; 60% annotations with correctness 0.85~0.9; 30% annotations with correctness 0.8~0.85	10%
U5/80	30% annotations with correctness 0.85~0.9; 40% annotations with correctness 0.8~0.85; 30% annotation with correctness 0.75~0.8	10%
U6/40	5% annotations with correctness 0.9~0.95; 20% annotations with correctness 0.85~0.9; 30% annotations with correctness 0.8~0.85; 30% annotation with correctness 0.75~0.8; 25% annotations with correctness 0.7~0.75	8%
U7/40	5% annotations with correctness 0.8~0.85; 15% annotations with correctness 0.75~0.8; 50% annotation with correctness 0.7~0.75; 30% annotations with correctness 0.6~0.7	7%
U8/30	10% annotations with correctness 0.75~0.8; 30% annotations with correctness 0.7~0.75; 60% annotation with correctness 0.6~0.7	3%
U9/30	All annotation are below 60% correct; 5 of them only contribute 5 or below annotations	2%

**Table 2:** Data deployment in pattern mining.

	Entities A group	Fre. Attr.	100% fre. Attr.	Max degree fre. associate Attr.	Frequent threshold
*s*1	12	24	24	24	0.95
*s*2	15	5	0	5	0.7
*s*3	10	20	2	15	0.5
*s*4	20	0	0	0	0.85
*s*5	16	49	0	1	0.7
*s*6	28	22	3	3	0.7
*s*7	36	24	3	3	0.7
*s*8	18	5	3	3	0.7

**Table 3:** Data deployment in ranking evaluation.

	Patterns	Annotations	Annotators	Entities
*c*1	49	5000	100	50
*c*2	100	10000	200	50
*c*3	196	20000	200	100
*c*4	285	30000	300	100
*c*5	400	40000	200	200

## References

[1] Gatterbauer W, Balazinska M, Khoussainova N, Suciu D (2009). Believe it or not: adding belief annotations to databases. *Proceedings of the VLDB Endowment*.

[2] Bhagwat D, Chiticariu L, Tan WC, Vijayvargiya G (2005). An annotation management system for relational databases. *VLDB Journal*.

[3] Eltabakh MY, Ouzzani M, Aref WG Managing biological data using bdbms.

[4] Wu AH, Tan ZJ, Wang W (2010). Annotation based query answer over inconsistent database. *Journal of Computer Science and Technology*.

[5] Wu AH, Tan ZJ, Wang W (2012). Query answer over inconsistent database with credible annotations. *Journal of Software*.

[6] Tweedie S, Ashburner M, Falls K (2009). FlyBase: enhancing drosophila gene ontology annotations. *Nucleic Acids Research*.

[7] Schneider M, Lane L, Boutet E (2009). The UniProtKB/Swiss-Prot knowledgebase and its plant proteome annotation program. *Journal of Proteomics*.

[8] Largillier T, Peyronnet G, Peyronnet S SpotRank: a robust voting system for social news websites.

[9] Wanas N, El-Saban M, Ashour H, Ammar W Automatic scoring of online discussion posts.

[10] http://www.w3.org/RDF/.

[11] Ai J, Meng XF (2009). C-Rank: a credibility evaluation method for deep web records. *Journal of Frontiers of Computer Science and Technology*.

[12] Brown J, Broderick AJ, Lee N (2007). Word of mouth communication within online communities: conceptualizing the online social network. *Journal of Interactive Marketing*.

[13] Ziegler CN, Lausen G (2005). Propagation models for trust and distrust in social networks. *Information Systems Frontiers*.

[14] Cheung M, Luo C, Sia C, Chen H (2009). Credibility of electronic word-of-mouth: informational and normative determinants of on-line consumer recommendations. *International Journal of Electronic Commerce*.

[15] Staddon J, Chow R Detecting reviewer bias through web-based association mining.

[16] Ghose A, Ipeirotis PG, Sundararajan A Opinion mining using econometrics: a case study on reputation systems.

[17] Gruhl D, Guha R, Kumar R, Novak J, Tomkins A The predictive power of online chatter.

[18] Hu M, Liu B Mining and summarizing customer reviews.

[19] Lee T, Bradlow ET (2007). Automatic construction of conjoint attributes and levels from online customer reviews. *The Wharton School Working Paper*.

[20] Liu B, Hu M, Cheng J Opinion observer: analyzing and comparing opinions on the web.

[21] Anthony D, Smith SW, Williamson T The quality of open source production: zealots and good samaritans in the case of Wikipedia.

[22] Hu M, Lim EP, Sun A, Lauw HW, Vuong BQ Measuring article quality in wikipedia: models and evaluation.

[23] Arighi CN, Roberts PM, Agarwal S (2011). BioCreative III interactive task: an overview. *BMC Bioinformatics*.

[24] Krallinger M, Vazquez M, Leitner F (2011). The protein-protein interaction tasks of BioCreative III: classification/ranking of articles and linking bio-ontology concepts to full text. *BMC Bioinformatics*.

[25] http://code.google.com/p/annotation-ontology/.

[26] Kwon D, Kim S, Shin SY, Wilbur WJ BioQRator: a web-based interactive biomedical literature curating system.

[27] Bui QC, Katrenko S, Sloot PMA (2011). A hybrid approach to extract protein-protein interactions. *Bioinformatics*.

[28] Abacha AB, Zweigenbaum P (2011). Automatic extraction of semantic relations between medical entities: a rule based approach. *Journal of Biomedical Semantics*.

[29] http://www.nactem.ac.uk/tsujii/GENIA/tagger/.

[30] http://arrowsmith.psych.uic.edu/cgi-bin/arrowsmith_uic/AnneOTate.cgi.

[31] http://www.ncbi.nlm.nih.gov/CBBresearch/Wilbur/IRET/PIE/index.html.

